# Habitat Capacity for Cougar Recolonization in the Upper Great Lakes Region

**DOI:** 10.1371/journal.pone.0112565

**Published:** 2014-11-12

**Authors:** Shawn T. O′Neil, Kasey C. Rahn, Joseph K. Bump

**Affiliations:** School of Forest Resources and Environmental Science, Michigan Technological University, Houghton, Michigan, United States of America; Institut Pluridisciplinaire Hubert Curien, France

## Abstract

**Background:**

Recent findings indicate that cougars (*Puma concolor*) are expanding their range into the midwestern United States. Confirmed reports of cougar in Michigan, Minnesota, and Wisconsin have increased dramatically in frequency during the last five years, leading to speculation that cougars may re-establish in the Upper Great Lakes (UGL) region, USA. Recent work showed favorable cougar habitat in northeastern Minnesota, suggesting that the northern forested regions of Michigan and Wisconsin may have similar potential. Recolonization of cougars in the UGL states would have important ecological, social, and political impacts that will require effective management.

**Methodology/Principal Findings:**

Using Geographic Information Systems (GIS), we extended a cougar habitat model to Michigan and Wisconsin and incorporated primary prey densities to estimate the capacity of the region to support cougars. Results suggest that approximately 39% (>58,000 km^2^) of the study area could support cougars, and that there is potential for a population of approximately 500 or more animals. An exploratory validation of this habitat model revealed strong association with 58 verified cougar locations occurring in the study area between 2008 and 2013.

**Conclusions/Significance:**

Spatially explicit information derived from this study could potentially lead to estimation of a viable population, delineation of possible cougar-human conflict areas, and the targeting of site locations for current monitoring. Understanding predator-prey interactions, interspecific competition, and human-wildlife relationships is becoming increasingly critical as top carnivores continue to recolonize the UGL region.

## Introduction

Cougars (*Puma concolor*) once spanned North and South America, ranging from south of the boreal forests to Patagonia [1], [2]. By the early twentieth century in the United States, human persecution, habitat degradation, and human expansion resulted in the extirpation of cougars from two-thirds of their historic range including eastern and midwestern America [3]. Cougars persisted only in the American west, where populations are increasing for the first time in nearly a century [4]. As a result of this increase, cougars are recolonizing portions of their former range. For example, natural recolonization, aided by changes in cougar protection and prey management, led to the return of viable populations in Wyoming and the Black Hills of South Dakota by 2000 [4]. Recolonization also occurred in the Badlands of North Dakota and in western Nebraska [5], [6].

Cougars, particularly young males, will travel hundreds of kilometers in search of new territory [7]–[9]. In 2011, one individual traveled more than 1,700 km from Minnesota to Connecticut, and may have traveled a straight-line distance of 2,500 km from the Black Hills to the East Coast [10]. In recent years, reports of cougar presence have been more frequent and widespread in the northern Midwest and Great Lakes States. Verified photograph and video evidence from automatic cameras, human-cougar encounters, DNA samples (scat, hair and blood) and track records was recorded each year in Michigan, Wisconsin, and Minnesota between 2008 and 2013. DNA analysis of samples suggested that a minimum of 6 individual cougars visited Wisconsin during this time period [11]. Evidence of cougar presence was also verified in Ontario, Canada [12]. Biologists believe confirmed cougar occurrences are young dispersing males from western populations, as evidenced by the presence of radio collars originating from western-based research and monitoring programs [10, Michigan Department of Natural Resources (DNR) *unpublished data*]. This increase in occurrence is consistent with the expectation that as populations rise in the American west, long-distance dispersals become more frequent, particularly among males [9], [13]. Cougars are now exploring much of the Midwest, ostensibly dispersing to expand to new territories, to increase mating opportunities, and to avoid overlap with existing home ranges [9], [13], [14].

The forests of northern Wisconsin and Michigan appear to exhibit high potential for cougar recolonization, due to favorable configurations of land cover, road density, abundant food resources, and relatively low human population densities [15], [16]. These same features have also shown to be important habitat features for gray wolves (*Canis lupus)* in this region [17]–[19]. Evidence of cougar range expansion has future conservation implications for the states of Michigan, Wisconsin, and Minnesota, which likely contain suitable habitat [20]. Despite little evidence of a breeding population [e.g. female cougars, kittens; but see [21], [22], these states are preparing for the emergence of a reproducing cougar population at some point in the near future [23]).

Potential cougar range expansion to the Upper Great Lakes (UGL) region raises important ecological questions and may require novel strategies to manage the species. Current research and management needs include mapping the geographic extent of potential cougar habitat in the UGL and estimating its capacity to support cougars. Researchers have modeled potential cougar habitat based on life history requirements to address questions of habitat extent where accurate data on the species' distribution are lacking [16], [20]. These models can be combined with prey densities to estimate an area's capacity for the species [24], [25]. Here, we address the need for spatially explicit cougar habitat data in Michigan and Wisconsin by extending an expert-assisted GIS spatial model [16], [20], [26]. We also provide an exploratory validation of the model, and estimate cougar capacity based on prey resources (white-tailed deer [*Odocoileus virginianus*]) and favorable land cover characteristics.

## Methods

### Study area

**Figure 1:**
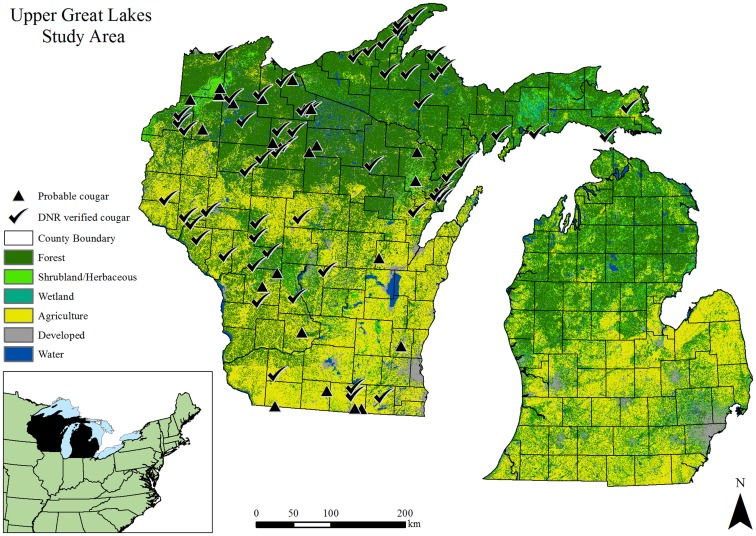
The study area for an analysis of cougar habitat and capacity for the Upper Great Lakes states, USA. Probable and verified cougar locations are represented from 1 January, 2008 to 1 June, 2013.

Our study area encompassed the states of Michigan and Wisconsin where DNR-confirmed cougar locations were widespread since 2008 (Fig. 1). Land cover types in the northern regions of both states are forest-dominated, with agriculture and human development becoming more predominant in the south. Forested land occurred over approximately 47% of the entire study area. Forests were generally of northern hardwood association, with common types including maple (*Acer* spp.) – birch (*Betula spp.*) – hemlock (*Tsuga canadensis*), aspen (*Populus* spp.) – birch (*Betula* spp.), and oak (*Quercus spp.*). Additional forest types included spruce-fir (*Picea* spp., *Abies balsamea*), and pine (*Pinus banksiana, P. resinosa, P. strobus*). Agricultural land use occurred on approximately 31% of the study area and generally consisted of wheat, corn, and soybean crops. Livestock grazing and dairy farming were common in more southern regions, particularly in Wisconsin. Other land cover types included shrubland/herbaceous, wetlands, and urban/developed (Fig. 1). Elevation ranged from 141 to 602 m and terrain was generally flat to hilly with an overall median slope estimate of 1.07° (Interquartile Range [IQR] = 0.34°–2.73°); however, more rugged terrain was present throughout the region and slopes reached 81.5° in some of these areas. The study area was characterized by abundant lakes and streams, with distance to water not exceeding 11 km (Median  = 0.35 km, IQR = 0.15–0.68 km). Human population density ≤5 persons/km^2^ characterized much of the northern regions of the study area, as opposed to the southern regions where population centers were more common and population densities commonly exceeded 100 persons/km^2^ in these areas.

### Overall Modeling Approach

We applied an expert-assisted spatial habitat model for cougars [16], [20], [26] to our study area. As a means of validation, we summarized predicted habitat around confirmed locations, and investigated the association between model-predicted cougar habitat values and confirmed cougar locations during the study period. We used the results of the habitat model to eliminate areas deemed unsuitable for cougars, incorporated estimates of deer densities and associated deer biomass within the UGL to investigate potential prey biomass, and subsequently combined potential landscape habitat characteristics with prey resources to generate a range of cougar capacity estimates for the UGL region.

### Cougar Habitat Model

We used previously established modeling framework and weights [16], [20], [26] to extend a cougar habitat model to the UGL. This model incorporated five, differently weighted parameters determined to be essential for cougar habitat: land cover, slope, human population density, distance to roads, and distance to water [26] (Table 1). All raster data generated by the habitat analysis were output to 30 m cell size in a GIS and we performed all spatial analysis in ArcGIS 10.1 (Environmental Systems Research Institute, Inc., Redlands, CA).

**Table 1:** Weights for attributes within variables used to model potential habitat suitability for cougars in the Upper Great Lakes states (Adapted from [26]).

Variable	Attribute	Weight (±S.E.)	Percent importance from highest ranking variable
Land cover	Mixed forest	1.92 (0.51)	100
	Deciduous forest	1.61 (0.37)	84
	Evergreen forest	1.59 (0.62)	83
	Shrublands	1.12 (0.85)	58
	Wetlands	0.67 (0.29)	35
	Grasslands	0.61 (0.47)	32
	Agricultural	0.28 (0.17)	15
	Barren/developed/other	0.19 (0.05)	10
Distance to paved roads	Long (>5 km)	1.43 (0.71)	100
	Medium (0.3–5 km)	0.88 (0.34)	62
	Short (<0.3 km)	0.69 (0.73)	48
Distance to water	Short (<1 km)	1.57 (0.41)	100
	Medium (1–5 km)	0.92 (0.27)	59
	Long (>5 km)	0.52 (0.27)	33
Human density	Low (<5 persons/km^2^)	2.28 (0.39)	100
	Medium-Low (6–10 persons/km^2^)	1.00 (0.18)	44
	Medium-High (11–19 persons/km^2^)	0.46 (0.27)	20
	High (>20 persons/km^2^)	0.25 (0.07)	11
Slope	Steep (>15°)	1.17 (0.54)	100
	Moderate (5–15°)	1.17 (0.41)	100
	Gentle (<5°)	0.66 (0.53)	56

To represent roads we used Tiger/line shapefiles acquired from the U.S. Census Bureau. We excluded non-paved roads from the analyses; remaining features represented paved roads that presumably impede cougar movement and/or increase risk of mortality [27]–[29]. We calculated distance to roads and reclassified the distances into three categories for use in the habitat model (Table 1). We calculated human population density (persons/km^2^) for each block group using 2010 U.S. Census Bureau data and grouped the results into four density classes (Table 1). We used the 1 arc-second (30 m spatial resolution) digital elevation model (DEM) from the National Elevation Dataset to calculate slopes (°) and reclassified the results into three categories (Table 1). We gathered rivers, streams, and lake features from the Michigan Geographic Data Library and from the ArcGIS Resource Center for Wisconsin. We retained all rivers, streams and water bodies assumed to hold water under normal, non-drought conditions (e.g. all lakes, rivers, and stream features including artificial paths, canals/ditches, intermittent streams, and perennial streams), calculated distance to these features, and reclassified distance to water into three categories (Table 1). We used the IFMAP/GAP Land Cover dataset [30] for Michigan and the National Land Cover Gap Analysis Project for Wisconsin. These datasets were comparable in that they allowed us to reclassify similar cover types into 8 final categories that were then weighted according to the initial habitat model [26] (Table 1).

**Figure 2:**
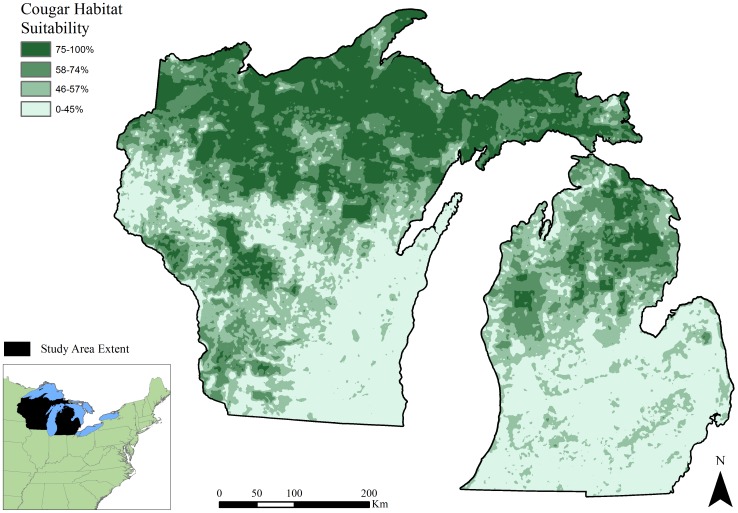
Cougar habitat rankings in the Upper Great Lakes region, USA. The cougar habitat model was generated based on expert-assisted variables and weights from LaRue [20], and LaRue and Nielsen [16], [26].

We weighted each variable in the model according to expert-based variable rankings and weights published previously [16], [26] (Table 1). Weights from this model were established via the Analytical Hierarchy Process (AHP) where experts reviewed all possible pairs of environmental attributes and assigned a rating based on each comparison [16], [26], [31]. Such analyses are useful for making *a priori* predictions in situations where empirical data are lacking, as is the case in our study area where until recently, cougars had not been confirmed present since the early 1900s [11], [32]. Next, we completed a weighted summation using the five variables and their respective weights (Table 2). We divided all pixels in the weighted sum raster by the maximum value that a pixel could receive, which resulted in a relative ranking for habitat potential [16]. Resulting pixel values ranged from 0 to 1, with 1 representing greatest habitat potential. To eliminate noise associated with pixel-scale variation, we smoothed the habitat raster using the mean neighborhood statistic within a 13.75 km^2^ circular moving window, corresponding to 1/4^th^ of the minimum home range size of a female cougar [9]. This size window retains a high level of resolution while also maintaining a realistic spatial scale for cougar perception of habitat, given that scale of selection is complex and likely varies depending on behaviors [33]. For ease of comparison, we scaled final habitat values into percentages and reclassified them into the same categories as the original cougar habitat model [16] (Fig. 2).

**Table 2:** Weights for each variable used in the calculation of potential habitat suitability for cougars in the Upper Great Lakes states (Modified from [26]).

Variable	Weights (S.E.)	Percent importance from highest ranking variable (land cover)
Land cover	1.84 (0.59)	100
Human density	1.22 (0.82)	66
Distance to paved roads	0.86 (0.45)	47
Slope	0.61 (0.56)	33
Distance to water	0.47 (0.26)	26

Weights were based on an Analytical Hierarchy Process analysis [31] and represent the relative importance of each variable to cougar habitat as established by a survey of experts [20], [26].

### Cougar Data and Model Validation

We reviewed current literature, archived reports, and press releases from the Michigan and Wisconsin DNRs, to document confirmed (i.e., verified evidence of) cougar occurrences in the study area from 1 January, 2008 to 1 June, 2013. Our review was supplemented by Rare Mammal Observations reported annually by the Wisconsin DNR [10], [34]–[37] and a confirmation database from the Michigan DNR (Michigan DNR, *unpublished data*). These sources of information included all known cougar confirmations documented in the study area since 2008 (Fig. 1). Dates were chosen because the first cougar confirmed in these states since the 1900s occurred in 2008, despite statewide collection of cougar reports from Wisconsin since 1991 [38]. Numbers of verified occurrences increased after 2008 [10], [34]–[37]. Verified evidence included tracks, photographs, scat, video, visual observation by wildlife officials, and DNA gathered from blood, scat, or hair. We recorded the date, township location, and type of evidence (photographs, tracks, scat, encounter, DNA) for each confirmation (Table 3, Fig. 1). Locations classified as “probable” occurrences by biologists based on available evidence [34] were included in Fig. 1 but not used for analysis.

**Table 3:** Confirmations of cougars by year, 1 January, 2008 to 1 June, 2013, in Michigan and Wisconsin, USA.

	Cougar confirmations			
Year	Michigan	Wisconsin	Probable sightings	Common methods^a^
2008	3	3	6	tracks, photo, DNA
2009	3	8	4	tracks, photo, observed (treed)
2010	1	6	5	photo, tracks, scat
2011	7	7	1	photo, tracks, scat (DNA), video
2012	6	13	9	photo, tracks, video, observed
2013^b^	0	1	NA	observed (treed)
Total	20	38	25	

a Ordered from most frequent to least frequent.

b Only includes confirmations reported through 1 June, 2013.

Confirmations are those verified by the Department of Natural Resources from either state as cougar (Michigan DNR, *unpublished data*; Wisconsin DNR, *unpublished reports*). Probable sightings were also reported in Wisconsin during the same time period.

We used the verified location data on the presence of cougars in Wisconsin and Michigan to assess the validity of the habitat model. We first summarized habitat model values around verified cougar locations at four spatial scales (i.e. buffers) to explore modeled habitat potential associated with known cougar space use. Four radial buffers were made around verified locations to represent the area potentially used by the observed animals within the time frame during which they were verified as present. Buffer radius distances were 1 km, 5 km, 10 km, and 25 km, respectively. These distances were chosen to cover a possible range of daily and weekly movement patterns, from smaller and more localized movements typically associated with resident cougars [28], [39], [40], to larger movements associated with long-distance dispersers [4], [8], [14]. We summarized mean modeled habitat values within these buffered areas. We then generated a random sample of “pseudo-absence” locations [41] to represent available habitat. We generated 100 random locations for every verified cougar locations over the study area, resulting in 5,800 availability samples [42]. Since no verifications occurred in the Lower Peninsula of Michigan, we limited this sample to Wisconsin and the Upper Peninsula of Michigan. We distributed the locations equally by county to ensure a spatially-balanced sample [42]. Then, for each location, we generated buffers using the same radius distances as the cougar locations and summarized habitat values within each buffer using GIS. To compare habitat values within used buffers to habitat values within available buffers, we assigned a binary indicator variable to each location (1 for used, 0 for available) and fit a resource selection function (RSF; [43], [44]) to these data via logistic regression [42]. Accordingly, the RSF assessed the influence that habitat values from our model had on the relative probability of cougar presence, allowing us to explore the validity of our habitat modeling approach. We performed the analysis for each buffer distance and thus fit 4 logistic regression models using ‘glm’ in R Version 3.0.0 (R Development Core Team, www.r-project.org, accessed 15 June 2013). We scaled the habitat model ranking to a percentage to simply model coefficient interpretation, assessed the resulting fit of models by comparing deviance residuals to the null model, and evaluated the ability of the habitat model to predict cougar occurrences using the Area Under Curve (AUC) statistic [45]. We calculated the AUC and its bootstrapped 95% confidence interval using the ‘pROC’ package in R [46].

### Prey Biomass

Carnivore abundance and density depends on prey availability and biomass as well as available space and favorable land cover. Predicting carnivore density based on estimates of prey biomass has been applied in wolves [17], [18], tigers (*Panthera tigris*) [47], and Canada lynx (*Lynx canadensis*) [24]. To estimate potential for cougar prey in the UGL, we used DNR estimates of deer density by deer management unit (DMU) in Michigan and Wisconsin [48], [49]. We assumed that white-tailed deer would be the primary prey for cougars in the region [50], [51]. To incorporate uncertainty in total deer biomass due to population sex/age structure across the UGL, we used three plausible sex/age structures (buck/doe/fawn) described for the northern Great Lakes and Ontario [52]: 40/30/30 (even/balanced), 25/40/35 (unbalanced, N. Wisconsin), and 15/50/35 (unbalanced, Michigan). We assumed the mean late-autumn deer size within these populations was 100 kg for bucks, 66 kg for does, and 33 kg for fawns [52]–[54]. Thus, deer biomass estimates (kg) for the three conceivable population structures were approximated within each DMU by the following:
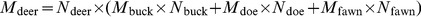
(1)where *N*_deer_ *= * estimated deer population/DMU, *M*_buck_  =  presumed average mass of an UGL white-tailed buck, *M*_doe_  =  presumed average mass of an UGL white-tailed doe, *M*_fawn_  =  presumed average mass of an UGL fawn, and *N* values for buck/doe/fawn were the proportion of deer in the sex/age class given a specified population structure. To estimate capacity for cougars in the study area, we first assumed habitat patches scoring <0.75 in the cougar habitat model would not support a population [16]. We subset the habitat model to only include values ≥0.75, restricted the DMUs to this subset, calculated the area in 100 km^2^ units for each DMU, and divided the deer biomass estimates by the area. Carbone and Gittleman [55] found that 10,000 kg/100 km^2^ could result in 0.94 cougars/100 km^2^; we multiplied deer biomass (10,000 kg/100 km^2^) by 0.94 to achieve the potential cougar density for each DMU. Thus, potential cougars/100 km^2^ depended on both deer density and available habitat based on favorable landscape characteristics.

Potential cougar density estimates (cougars/100 km^2^) corresponding to the three aforementioned deer population structures were summed across the study area. To incorporate geographic variation, we averaged potential cougar density across all available habitats using a 100 km^2^ circular assessment window. While densities of up to 13 cougars/100 km^2^ have been reported in other locations [56], such densities are uncommon [33], [57], [58]. Given the potential for cougar-human conflicts leading to higher risk of mortality and competition with wolves and other predators for prey [59], we considered it unlikely for population densities to exceed 3 cougars/100 km^2^ in the UGL. Thus, despite high estimates of prey biomass in some areas, we limited the maximum potential cougar density to 3/100 km^2^. To incorporate additional uncertainty, we also carried out the analysis using maximum potential cougar densities of 2/100 km^2^ and 1/100 km^2^.

## Results

### Cougar Habitat Model

Our model indicated high potential for cougar habitat in Michigan and Wisconsin. Cougar habitat values ranged from 0.27 to 0.92 (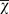 = 0.56, SD = 0.15). Assuming the value ≥0.75 is a conservative estimate of suitable habitat [16] at least 39% (>58,000 km^2^) of our total study area contained suitable cougar habitat. Habitat areas were generally contiguous and concentrated throughout the forested regions of northern Wisconsin, much of the Upper Peninsula of Michigan, and the northern Lower Peninsula of Michigan (Fig. 2). The largest contiguous patch of suitable habitat occurred in northern Wisconsin and the Upper Peninsula. This area contained 85% (49,216 km^2^) of all habitat receiving scores of 0.75 or higher. According to our model, patches appeared to decrease in frequency and size and were evidently more fragmented at southern latitudes. Less suitable cougar habitat (suitability values <0.75) often coincided with higher human population densities and agriculture-dominated landscapes; these areas were concentrated throughout the south and south-central regions of the study area (Fig. 2).

Cougar confirmations from the DNR in Michigan and Wisconsin totaled 58 from 1 January, 2008 to 1 June, 2013 (38 and 20 in Wisconsin and Michigan, respectively; Table 3). Model validation results suggested that the habitat model was effective in identifying areas of suitable cougar habitat. Habitat values summarized around cougar locations exhibited a wide range of habitat potentially used by cougars in the UGL, but were generally consistent with the expectation that verified locations would occur within higher-ranked vs. lower-ranked habitat. Observed habitat values within buffered areas ranged from 0.27 to 1.00 depending on buffer size (Table 4) and occurred within higher-ranked habitat than the mean cougar habitat ranking for the study area (0.56). Our cougar habitat model was a significant predictor of the relative probability of cougar occurrence at each buffer distance (Table 4). The RSF deviance residuals at each scale improved upon the null model and odds ratios associated with the habitat predictor were significantly greater than one (Table 4) indicating that the odds of an occurrence increased with increases in habitat ranking. The strength of this effect and the model's predictive performance (AUC) both increased slightly with larger buffer sizes (Table 4). At each scale, the odds of a cougar occurrence increased by 2–6% with each percent increase in cougar habitat ranking (Table 4).

**Table 4:** Modelled cougar habitat values (0–100%) association with 58 cougar occurrences in Michigan and Wisconsin, USA at 4 spatial scales between 2008 and 2013.

Buffer radius	Range	Mean (± SD)	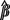 Habitat	Odds ratio (95% CI)	AUC (95% CI)
1 km	33–90	64±15	0.033	1.03 (1.02–1.05)	0.66 (0.59–0.72)
5 km	29–92	64±15	0.034	1.03 (1.02–1.05)	0.66 (0.59–0.72)
10 km	29–94	65±15	0.038	1.04 (1.02–1.06)	0.67 (0.60–0.73)
25 km	27–100	64±15	0.042	1.04 (1.02–1.06)	0.68 (0.62–0.74)

Coefficients, odds ratios, and area under curve (AUC) statistics were generated by logistic regression models linking modeled habitat values to verified occurrences.

### Cougar Capacity

**Figure 3:**
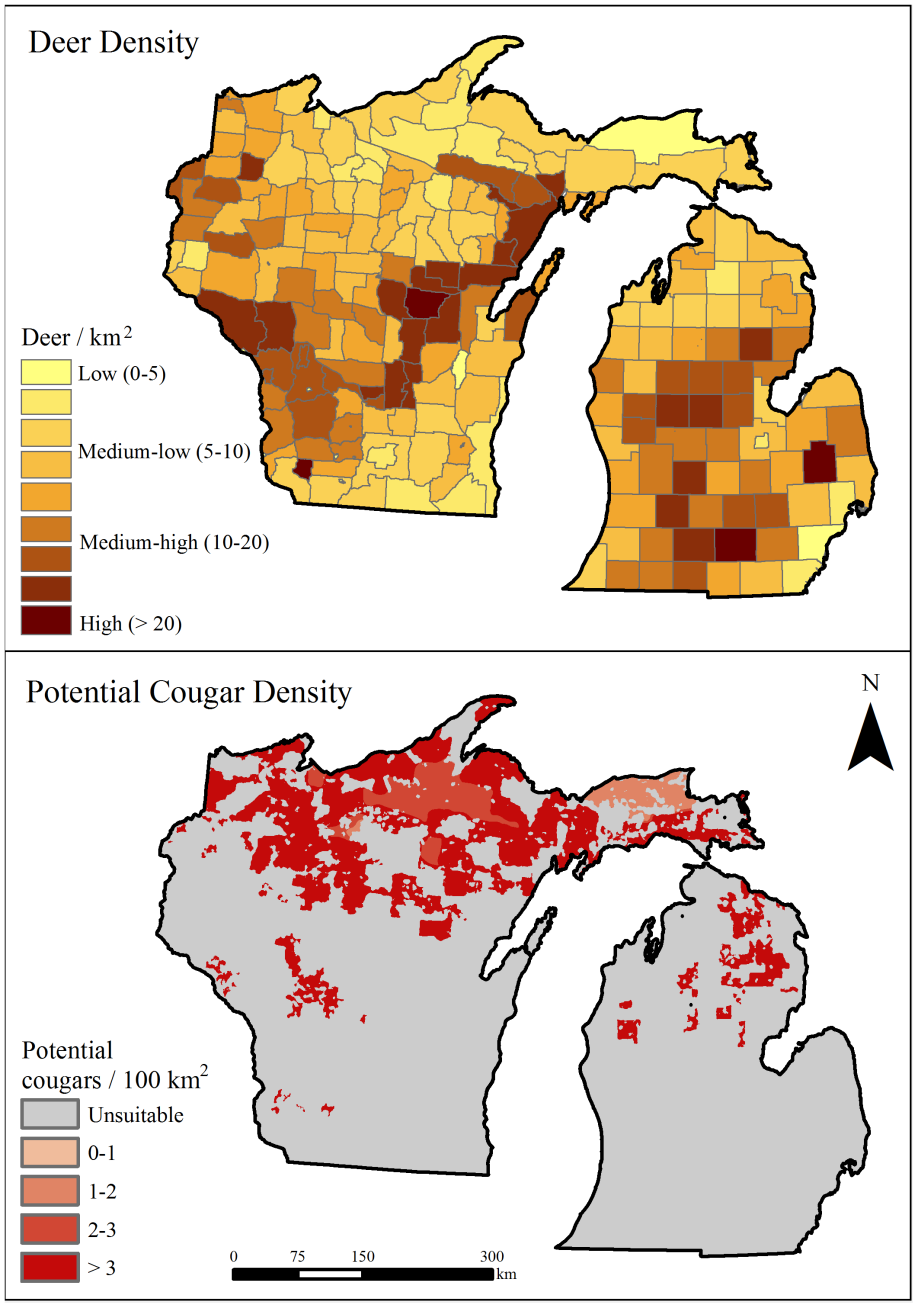
Prey densities and cougar capacity in the Upper Great Lakes region, USA. **a**) Whitetail deer density estimates were based on best available information from state natural resource management agencies [48], [49]. **b**) Geographic variation in cougar capacity based on our most conservative potential deer biomass estimates. Deer biomass estimates were generally high enough to support >3 cougars/100 km^2^ across much of the study area.

Deer density estimates per DMU ranged from 0.5 to >20 deer/km^2^ (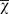 = 11.1, SD = 6.1; Fig. 3a). Using these estimates and three potential age/sex population structures, deer biomass per DMU could conceivably range from approximately 3,000 kg/100 km^2^ (unbalanced age structure, lowest deer density) to over 200,000 kg/100 km^2^ (balanced age structure, highest deer density). After restricting the DMUs to favorable cougar habitat, the mean deer biomass estimates per DMU within potential cougar habitat were approximately 10,000–165,000 kg/100 km^2^ depending on the age/sex structure applied (Table 5). Thus, we estimated that prey biomass within potential cougar habitat was geographically variable and could support up to 15 cougars/100 km^2^ (Fig. 3b). However, we also assumed that >3 cougars/100 km^2^ anywhere within the study area was unrealistic despite high deer densities in some areas. Using three different maximum viable densities (1, 2, and 3 cougars/100 km^2^) and allowing lower estimates to depend on approximations of deer biomass where deer densities were low, we calculated that available resources could sustain 582 to 1,677 cougars within favorable habitat.

**Table 5:** Estimates of white-tailed deer biomass within deer management units for Michigan and Wisconsin (the Upper Great Lakes region, or UGL); estimates are based on three conceivable age/sex structures for white-tailed deer and are restricted to favorable habitat as indicated by cougar habitat model values ≥0.75.

Bucks/does/fawns	Range (kg/DMU)	Mean (kg/DMU)	SD (kg/DMU)	Total biomass (kg)
40/30/30	12,494–165,450	76,189	31,812	7.70×10^6^
25/40/35	11,284–149,427	68,810	28,731	6.95×10^6^
15/50/35	10,674–141,357	65,094	27,179	6.57×10^6^

## Discussion

Our extension of a habitat model for cougars [16], [20], [26] suggests that Michigan and Wisconsin contain >58,000 km^2^ of potential cougar habitat. Based on potential deer biomass estimates, more than 500 cougars could inhabit this overall area. For comparison, cougar range in Washington, USA covers 51% of the state (88,500 km^2^) and consistently supports approximately 2,000 cougars [60], and estimates from several models in the northeastern U.S. suggested potential for 322–2,535 cougars depending on the area considered suitable [61]. The northern half of our study area is characterized by dense forest, low human population and road densities, abundant water resources, and diverse topography. Large expanses of wild land exist that provide contiguous habitat characterized by forested landscapes which support high densities of white-tailed deer. Confirmed location data and associated radio-collar and DNA evidence suggest that multiple cougars occurred in this area between January 2008 and early 2013.

Our investigation of habitat capacity should not be viewed as an attempt to predict the precise distribution and/or exact size of a potential cougar population. Although it would be possible to speculate based on the models we presented (e.g. [18]), the nature of recolonization (i.e. breeding range selection, population size, geographic distribution) will likely depend on additional factors that we could not quantify such as future changes in management, public acceptance [62], the presence and density of competing predators, potential disease components, migratory behavior in deer [63] and other dynamics of prey populations. Both states have experienced similar conservation challenges as wolves have recolonized and recovered in the region [64], [65]. Our model validation analysis is exploratory in nature, as the best available information for cougar presence in the UGL is primarily based on incidental, verified observations (Table 3); these location data are assumed to be associated with transient cougars from western populations [13]. Detection and verification probability may vary geographically, and this variation can bias information on cougar presence. Automatic cameras are not evenly spread across the landscape, and are likely underused on large blocks of public lands that contain some of the best cougar habitat in the region. In addition, resource selection by dispersing, transient animals may differ from that of resident individuals [26], [66], perhaps with particular regard to human development and activity [15], [33]. These reasons likely contributed to our habitat model's relatively low ability to predict cougar occurrences on our study site (model validation AUC scores between 0.59 and 0.74; Table 4). Without knowledge of the specific nature of detection probability across the study site, modeling cougar distribution (e.g. probability and/or likelihood of occurrence) based on presence-only data would require strong assumptions [67], [68]. Similarly, it would not be appropriate to base estimates of cougar distribution entirely on locations associated from animals assumed to be non-resident individuals. As such, although our validation used the best available information to provide support for our habitat model, information on cougar habitat use in the UGL remains limited and may not be adequate for making predictions. Given that our objective was to model habitat potential for a future resident population, the expert-assisted model that we implemented is preferable because it only makes assumptions about the most general habitat requirements for cougars. We showed that verified cougar locations in the UGL were consistent with our model, which further suggests that areas of modeled suitable habitat may be able to support a viable cougar population in the future.

Recolonization and recovery of cougars to former ranges such as the UGL will likely require active and adaptive management at both state and federal levels. Natural recolonization is likely to occur eventually [69], providing states with favorable cougar habitat the opportunity to prepare. Public attitudes and their associated influences on public policy are important determinants of large carnivore population viability [62]. Recent evidence suggests that people may be more neutral and are consequently more impressionable in regions where cougars have previously been irrelevant [70], but that humans can be accepting and supportive of cougars, particularly if they have access to good information and are knowledgeable about cougar ecology [62], [69], [71]. These findings have implications for areas of potential cougar recolonization because there may be a relatively small window of opportunity for outreach and education programs to promote awareness and shape public opinion, as overcoming limited trust once it is instilled is difficult [70]. Large carnivore populations can be polarizing, in part because management tools are often controversial. The appropriateness and ethics of recreational hunting and population control of these animals are hotly debated [72], [73] and understanding how public attitudes are influenced by these actions is complicated [74]. Consequently, a natural first step toward preparing for a cougar recolonization would be investigating social acceptance and potential human tolerance of cougars in the UGL states. Public education and outreach could positively shape public opinions and help to avoid agency mistrust [69]. The state of Wisconsin has drafted a management protocol in the event that a breeding population establishes [36]. Additionally, all confirmed cougar observations are publicly available [11]. Anticipating recolonization prior to its occurrence can improve future management and could lay the groundwork for a strong, collaborative cougar conservation program regionally.

In the event that a population becomes established in the UGL, state DNR agencies will need to address concerns of human safety, pet safety, and depredation of livestock [15], [75], as well as develop long-term monitoring programs [76]. Strategies necessary to manage a cougar population will likely include plans for education and outreach associated with human safety concerns, potential compensation for livestock losses, mitigation strategies for conflicts with farmers/ranchers, and discussion of possible harvest scenarios [15], [75]. Long-term research investigating cougar behavior, habitat use, prey selection, genetic structure of the population, competition with other predators, and impacts on white-tailed deer could eventually be warranted. The addition of another carnivore to the current predator guild in the UGL states could be politically challenging, yet long-term ecological benefits of a viable cougar population [77] could be realized under effective conservation planning scenarios.
